# Prior Cervical Cancer Screening, Social Determinants of Health, and HIV Status Among Patients at the Cancer Disease Hospital, Zambia

**DOI:** 10.21203/rs.3.rs-10345811/v1

**Published:** 2026-07-22

**Authors:** Graciela M. Nogueras Gonzalez, Susan Msadabwe, Bernadette Njala, Dyness Sakala, Mwando Chitula, Jane R. Montealegre, Susan K. Peterson, Anna E. Coghill, Paul Kamfwa, Elizabeth Y. Chiao, Lilie L. Lin

**Affiliations:** The University of Texas MD Anderson Cancer Center; Cancer Diseases Hospital; Cancer Diseases Hospital; Cancer Diseases Hospital; Cancer Diseases Hospital; The University of Texas MD Anderson Cancer Center; The University of Texas MD Anderson Cancer Center; Moffit Cancer Center; Cancer Diseases Hospital; The University of Texas MD Anderson Cancer Center; The University of Texas MD Anderson Cancer Center

**Keywords:** Cervical Cancer Screening, Social Determinants of Health, Uterine Cervical Neoplasms, Epidemiology, Cross-Sectional Studies, Health Services Research, Developing Countries, HIV

## Abstract

**Background:**

Cervical cancer screening uptake remains very low in sub-Saharan Africa, particularly in Zambia, where limited access results in high cervical cancer mortality. Social determinants of health (SDOH) are among the factors influencing low screening rates. Women living with HIV (WLHIV) are especially vulnerable due to additional risk factors. Although Zambia has expanded screening programs into HIV clinics, the impact of SDOH on screening uptake is not well understood. This study investigated the relationship between prior cervical cancer screening, SDOH, and HIV amongst diagnosed cervical cancer patients at the Cancer Disease Hospital (CDH) in Zambia.

**Methods:**

Newly diagnosed, untreated patients aged ≥ 18 years who presented to CDH for treatment for histologically confirmed cervical cancer between 2022 and 2026 were enrolled in a prospective, IRB-approved cohort and responded to a health-related social needs questionnaire. We constructed an SDOH score using the 5 Healthy People 2030 domains: economic stability, education, health/substance use, neighborhood and built environment, and social and community context. The association between prior cervical cancer screening and SDOH score was analyzed using logistic regression models overall and stratified by HIV status.

**Results:**

A total of 464 patients were enrolled (median age 50 y [IQR:43–57]). Of these patients, 215 (46.3%) reported having undergone prior screening, 178 (46.4%) had advanced-stage cancer (stage III/IV), and 251 (54.1%) were WLHIV. Early cancer stage (stage I/II) was associated with having undergone prior screening (*p* = 0.017). A higher proportion of screened individuals were 50–59 years of age (*p* = 0.001) compared to other age groups. Having education about and knowledge of cervical cancer was associated on unadjusted analysis with prior screening. On adjusted analysis for HIV status and age, patients with lower SDOH scores (indicating less disadvantage) had 2.21 times higher odds of having undergone prior screening than women with higher SDOH scores (*p* < 0.001). Additionally, WLHIV had 2.72 times higher odds of having undergone prior screening compared to women without HIV (*p* < 0.001).

**Conclusions:**

Age, SDOH, and HIV status were associated with having undergone prior cervical cancer screening. Further research is warranted to understand barriers to screening and to explore the acceptance of screening among women without HIV in Zambia.

## Background

Cervical cancer is a major public health concern in sub-Saharan Africa, where it is one of the leading causes of cancer-related mortality among women. Zambia, in particular, faces a disproportionately high burden of cervical cancer due to limited access to screening and early treatment services. For both the general population and women living with HIV (WLHIV), the World Health Organization (WHO) recommends human papillomavirus (HPV)-based testing as the primary cervical cancer screening test. WHO suggests regular screenings every 5 to 10 years for the general population and every 3 to 5 years for WLHIV. (1) Among the three main screening methods available—cytology or Papanicolaou testing, HPV testing, and visual inspection with acetic acid (VIA)—VIA is the most widely used in low- and middle-income countries (LMICs) such as those in Africa because it is low-cost, gives immediate results, and supports “screen-and-treat” models. However, VIA has several limitations, including low accuracy, high overtreatment rates (particularly in WLHIV), the need for provider training, and inconsistent linkage to treatment.(2) Despite the availability of effective screening methods, uptake of cervical cancer screening in LMICs remains critically low. A recent umbrella review reported that the average cervical cancer screening prevalence in Africa is only 8.9%, with significant disparities across countries and populations.(3) Among WLHIV, additional risk factors such as low CD4 cell counts, having 5 or more abortions, and vaginal wall abnormalities increase vulnerability to cervical cancer.(4)

Screening uptake is influenced by a range of social determinants of health (SDOH) factors. Women who are older, more educated, employed, wealthier, exposed to media (read a newspaper, listen to the radio, or watch television), or live in urban areas are significantly more likely to undergo screening.(5, 6) In Zambia, health system and infrastructure limitations—including reliance on donor funding, supply chain disruptions, limited diagnostic facilities, referral delays, and a shortage of trained healthcare providers—are also major barriers to effective screening(7) and contribute to late-stage diagnoses and poor outcomes.(8, 9) Economic barriers also play a critical role. The cost of travel, screening, and treatment prevents many women from accessing services, especially in rural areas.(10) Cultural and social factors, including stigma, misconceptions, and lack of spousal support, further discourage screening participation. For example, cancer is often perceived as a death sentence or contagious, leading to fear and avoidance of screening.(11, 12) Individual-level challenges such as low awareness, fear of pain, and embarrassment during pelvic exams are also common deterrents.(13, 14) Finally, lack of formal education on sexual health and cancer screening literacy remains a persistent issue.(15)

Despite the expansion of cervical cancer screening programs into HIV clinics in Zambia and recognition of structural and individual-level barriers, it remains unclear how these factors affect screening rates among women who are later diagnosed with cervical cancer, particularly among WLHIV. To address this gap, we conducted a cross-sectional study of women recently diagnosed with cervical cancer at the Cancer Disease Hospital (CDH) in Lusaka, Zambia’s national referral center for cancer care, to evaluate the prevalence and determinants of prior screening. By examining individual, social, and systemic factors associated with screening history, this study aims to inform context-specific strategies to enhance screening uptake and improve outcomes for women in Zambia.

## Methods

### Study Design and Setting

This cross-sectional analysis was conducted within an ongoing prospective cohort study of women newly diagnosed with cervical cancer at CDH. Although participants were enrolled prospectively, the present analysis evaluates self-reported prior cervical cancer screening and associated factors at the time of study enrollment. CDH is the national referral center for oncology care and the only facility in Zambia providing comprehensive cancer treatment, receiving patients from across the country. As a result, the study population reflects women who present for treatment of cervical cancer, many of whom are diagnosed at advanced stages following gaps in screening and early detection. This setting provides a unique opportunity to examine screening histories and barriers among women who have progressed to cancer diagnosis despite the availability of screening programs.

### Study Population

Using convenience sampling, the study enrolled eligible participants presenting from June 2022 to May 2026 with newly diagnosed, histologically confirmed FIGO stage IB-IIIC cervical cancer who were receiving chemoradiotherapy. Eligible participants were aged 18 years and older, provided informed consent, had a Karnofsky performance status score of 70 or higher, and a hemoglobin level more than 9 g/dL. For WLHIV, additional inclusion criteria were receipt of a stable antiretroviral therapy regimen for at least 6 months and viral suppression. Exclusion criteria included prior receipt of chemotherapy, use of any medications that could affect the immune system (other than antiretroviral therapy), and evidence of hydronephrosis, para-aortic lymphadenopathy, or pelvic side wall disease, as these factors could impact ability to complete chemoradiotherapy.

### Data Collection

We adapted a structured health-related social needs questionnaire from one developed by the US Centers for Medicare and Medicaid Services to capture information on SDOH indicators (economic stability, education, health/substance use, neighborhood and built environment, and social and community context)(16, 17). The questionnaire was translated into the 4 most common languages spoken in Zambia (Tonga, Nyanga, Lozi, and Bemba) and then back-translated into English to verify the translations. The questionnaire was administered by trained research assistants; participants could self-complete the questionnaire or have the questionnaire read aloud when literacy barriers were present (Supplemental Material). Data on demographics (age, marital status, and province of residence) and clinical characteristics (HIV status, and cancer stage at diagnosis) were extracted from the patients’ medical records. The primary outcome was prior cervical cancer screening, defined as self-reported history of undergoing any cervical cancer screening test before diagnosis.

### SDOH Measures

We constructed an SDOH score using 5 domains identified by the US Department of Health and Human Services’ Healthy People 2030 initiative—economic stability, education, health/substance use, neighborhood and built environment, and social and community context—as a framework.(18) Following the methodology used by Menza et al.,(19) we chose 2 items from our modified questionnaire that best mapped to each of the 5 SDOH domains (Table 1). Housing instability, a component of the “neighborhood and built environment” domain, was assessed by summing 7 house characteristics into a score ranging from 0 to 7: type of house, type of roof, source of drinking water, type of energy source, location of the kitchen, problems with pests, and problems with electricity. This approach was used to capture the multidimensional nature of housing conditions assessed in the questionnaire, which are not adequately represented by a single indicator and may differentially influence health and healthcare access. To calculate the SDOH score, each item was scored as 0 or 1, with 0 indicating no or less disadvantage, and 1 indicating more disadvantage. The sum of the items for each woman yielded an SDOH score ranging from 0 to 10, where higher scores indicated greater social, environmental, or economic disadvantage (Table 1). From this continuous score, we derived a categorical variable based on the median SDOH scores: women with scores of 0 to 4 were classified as having lower disadvantage, and those with scores of 5 to 10 as having higher disadvantage.

### Statistical Analysis

We used descriptive statistics to summarize participant characteristics. Continuous variables were reported as medians with interquartile ranges (IQR), and categorical variables as frequencies and percentages. Bivariate analyses were conducted using chi-square tests for categorical variables and Wilcoxon rank-sum tests for continuous variables to compare individuals who were previously screened versus unscreened individuals.

Adjusted logistic regression was used to identify independent predictors of prior screening. Variables with *p* < 0.05 in univariable analyses were considered for the multivariable model; we applied backward stepwise elimination (retention threshold *p* ≤ 0.10). We reported odds ratios (ORs) with 95% confidence intervals (CIs); statistical significance was set at *p* < 0.05.

Given that cervical cancer screening is done in HIV clinics in Zambia, and half of the patients in the cohort were WLHIV, we conducted analyses stratified by HIV status a priori to assess potential effect modification of the associations between sociodemographic factors, SDOH, and prior cervical cancer screening.

## Results

The study enrolled 464 women with histologically confirmed cervical cancer. The median age of the participants was 50 years (IQR: 43–57) (Table 2). Among the participants, 46.3% (*n* = 215) reported having undergone prior cervical cancer screening. Although there was no significant difference in median age between screened and unscreened participants (*p* = 0.050), a higher proportion of screened individuals were 50–59 years of age (*p* = 0.001) compared to other age groups (29–39, 40–49, and 60–78). Patients traveled to CDH from all provinces of Zambia, but the largest number was from Lusaka (28.7%). About half of the women (53.6%) in our cohort were diagnosed with early-stage disease (stage I, 11.5%; stage II, 42.2%), and 46.4% were diagnosed with advanced-stage disease (stage III, 39.1%; stage IV, 7.3%). Early-stage disease was more common among screened women (51.0%) than was advanced-stage disease (38.8%, *p* = 0.017). A majority of the women in the cohort were WLHIV (54.1%). Additionally, WLHIV constituted 58.6% of the participants who had undergone cervical cancer screening, compared to 41.4% in the unscreened group (*p* < 0.001) (Table 2).

SDOH scores were calculated for each participant using the questions presented in Table 1 (0–4 indicators: lower disadvantage, and 5–10 indicators: higher disadvantage). More unscreened women had SDOH scores indicating higher disadvantage (62.9%) compared to screened women (37.8%; *p* < 0.001). Regarding education, 62.9% of the screened women had secondary or tertiary education (vs. 37.1% unscreened), and more screened women reported having knowledge about cervical cancer (74.3%) compared to unscreened women (25.7%; *p* < 0.001 for both). Urban residence was positively associated with screening uptake (*p* = 0.002), with 53.6% of screened participants residing in urban areas versus 46.4% among the unscreened. Additionally, a higher proportion of unscreened women than screened women reported being unable to do their day-to-day activities on their own (62.4% vs 37.6%, respectively; *p* = 0.022) (Table 3).

In the adjusted models (Table 4), women aged 60–78 were less likely to be unscreened than were those aged 50–59 (OR = 0.41, 95% CI: 0.23–0.73, *p* = 0.003), while age groups 21–39 and 40–49 were not significantly associated with screening status. WLHIV were more likely to have been screened compared to HIV-negative women (OR = 2.72, 95% CI: 1.83–4.05, *p* < 0.001). Women with lower SDOH scores (i.e., less disadvantage) were more likely to be screened than those with higher SDOH scores (OR = 2.21, 95% CI: 1.48–3.28, *p* < 0.001).

Because HIV status was strongly associated with prior screening in the overall cohort and more than half of participants were WLHIV, we next examined whether associations between SDOH indicators and screening differed by HIV status (Table 5). The median age of WLHIV was lower than that of HIV-negative women (48 years [IQR: 42–54] vs. 52 years [IQR: 43–60]; *p* = 0.003). A higher proportion of WLHIV were 40–49 years of age (62.3%) compared with HIV-negative women (37.7%; *p* = 0.002). A lower proportion of WLHIV than HIV-negative women were married (45.4% vs. 54.6%; *p* < 0.001). There were no significant differences in cancer stage between WLHIV and HIV-negative women (*p* > 0.05). A higher proportion of WLHIV were from Lusaka (63.2%) and Copperbelt (62.0%) compared with HIV-negative women (36.8% and 38.0%; *p* = 0.027).

Most WLHIV reported SDOH scores of 0–4 (60.2%), which was significantly higher than the rate in HIV-negative women (39.8%; *p* = 0.030). Regarding education, 62.9% of WLHIV had secondary or tertiary education (vs. 37.1% of HIV-negative women), and 66.7% of WLHIV reported knowledge of cervical cancer (vs. 33.3% of HIV-negative women) (*p* < 0.05 for both). A higher proportion of WLHIV reported consuming alcohol compared with HIV-negative women (67.3% vs. 32.7%, respectively; *p* = 0.001). Urban residence was positively associated with HIV status, with 62.3% of WLHIV and 37.7% of HIV-negative women residing in urban areas (*p* < 0.001), **Supplemental Table 1**.

In adjusted models stratified by HIV status (Table 6), among HIV-negative women, those aged 60–78 were less likely to be unscreened compared to those aged 50–59 (OR = 0.31, 95% CI: 0.13–0.78, *p* = 0.012), while ages 21–39 and 40–49 showed no significant differences in screening status. HIV-negative women with lower SDOH scores were more likely to have been screened than those with higher SDOH scores (OR = 2.18, 95% CI: 1.13–4.17, *p* = 0.019). No significant association was found for marital status.

Similarly, in the adjusted model for WLHIV, women with lower SDOH scores were more likely to have been screened than were those with higher SDOH scores (OR = 2.15, 95% CI: 1.25–3.69, *p* = 0.006). Married WLHIV were less likely to be to have been screened than were those unmarried WLHIV (OR = 0.57, 95% CI: 0.33–0.97, *p* = 0.040). No significant associations were found for age and province.

## Discussion

To our knowledge, few studies in LMIC settings have prospectively linked prior cervical cancer screening history directly to disease stage at carcinoma diagnosis and SDOH. Among our cohort of 407 women with newly diagnosed cervical cancer at the CDH in Lusaka, Zambia, fewer than half (45.2%) reported any prior screening. Screening was more common in women aged 50–59, WLHIV, women with secondary or tertiary education, those with knowledge about cervical cancer, and those residing in urban areas. Conversely, greater social disadvantage (i.e., higher SDOH score) and rurality were associated with being unscreened. As expected, prior screening was associated with a higher likelihood of early-stage diagnosis.

Higher screening rates among WLHIV likely reflect more frequent healthcare encounters, integration of screening within HIV care, and provider-initiated prompts.(20, 21)In contrast, HIV-negative women may have fewer routine contacts with the health system and must often rely on outreach or primary care settings, where screening opportunities are less consistent. These observations highlight the opportunity to leverage HIV care infrastructure to expand screening for all women—regardless of HIV status—by co-locating services, employing shared patient navigators, and establishing standardized referral pathways.(22–24)

Zambia established a Cervical Cancer Prevention Program in 2006, a national initiative offering free screening to all eligible women attending clinics. Additionally, the Go Further public-private partnership—supported by the US President’s Emergency Plan for AIDS Relief and other organizations—aims to reduce cervical cancer incidence by providing screening and treatment services through HIV clinics in Zambia and other sub-Saharan African countries. Nevertheless, despite the availability of free screening for both WLHIV and HIV-negative women, many still present to CDH with advanced-stage disease.

The SDOH gradient observed—where women experiencing greater social disadvantage were less likely to be screened—highlights the pervasive role of structural and socioeconomic barriers to screening. Education and knowledge facilitate preventive care, while housing instability and neighborhood disadvantage hinder access. Similar barriers have been documented in Zambia and other low-resource settings.(12, 25, 26) Urban residence was positively associated with screening uptake, likely due to better information dissemination and service availability, although gaps in awareness persist.(27) In sub-Saharan Africa, screening rates for various cancers, including cervical and breast cancer, are generally higher in urban areas. (28–30) Urban residency often correlates with higher socioeconomic status, which is associated with higher screening rates. A study on cervical precancer screening across Ethiopia, Tanzania, Zambia, and Zimbabwe found significant inequalities in screening probabilities, with urban, older, more educated, and wealthier women having higher screening rates.(31) Together, these findings suggest that urban residency, combined with other socioeconomic factors, significantly influences screening uptake.

Stratified analyses by HIV status demonstrated that the SDOH gradient in screening uptake persisted in both WLHIV and HIV-negative women, with lower social disadvantage consistently associated with higher odds of prior screening. The similarity of these associations across strata suggests that, within this cohort of women diagnosed with invasive cervical cancer, WLHIV did not experience fundamentally different structural or socioeconomic barriers to screening compared with HIV-negative women. The HIV-stratified analyses therefore serve primarily to confirm the robustness of the SDOH–screening association across HIV status, rather than to identify distinct HIV-specific screening barriers within this cohort.

Differences in age patterns between strata, particularly higher screening uptake among older HIV-negative women, may reflect cohort effects, differential healthcare contact, or limited statistical power to detect age effects among WLHIV. Importantly, the absence of large differences by HIV status should not be interpreted as the absence of HIV-specific barriers; rather, it suggests that shared structural and social determinants may dominate screening behavior across groups in this setting. A screening uptake rate of 45% among women presenting for cancer care represents a significant missed opportunity for prevention and early detection. The association between prior screening and earlier stage at diagnosis underscores the clinical importance of screening in enabling disease detection at more treatable stages, consistent with national and global strategies to reduce cervical cancer mortality through timely screening and intervention.(32–34) This relationship aligns with existing evidence that screening accelerates diagnosis and improves outcomes. For instance, a Zambian study introducing a breast specialty service within a primary healthcare facility reported increased early-stage breast cancer diagnoses and improved survival rates.(10) These findings reinforce that accessible, efficient screening services can substantially enhance early detection and clinical outcomes.

### Strengths and limitations

Strengths of this study include the large sample with histologically confirmed diagnoses, systematic characterization of SDOH indicators, and adjusted analyses including stage stratification. Our approach is unique in that it integrated a structured SDOH questionnaire with standard epidemiologic data, enabling the assessment of prior screening and identification of SDOH barriers associated with underscreening. This approach aimed to inform targeted interventions for early detection and mortality reduction. Limitations include potential recall and social desirability bias in self-reported prior screening, cross-sectional design limiting causal inference, hospital-based sampling (which may not capture women not presenting to tertiary care), and possible residual confounding (e.g., unmeasured access factors, provider practices). Stratified analyses may have been underpowered to detect modest differences between WLHIV and HIV-negative women, particularly for age and marital status, and null findings should be interpreted cautiously.

Several important barriers to screening among WLHIV were not captured in the current analysis. Although the SDOH instrument assessed structural and socioeconomic conditions, it did not include measures of HIV-related stigma, fear of disclosure, or discrimination, which are known to influence care-seeking behavior and screening uptake among WLHIV. The absence of these measures may partly explain the similarity of associations observed across HIV strata. Additionally, stratified analyses may have been underpowered to detect modest differences between groups.

## Conclusion

In this Zambian cohort, prior screening was associated with earlier-stage diagnosis, and screening uptake was highest among WLHIV, urban residents, and women with lower social disadvantage, higher levels of education, and greater knowledge about cervical cancer. HIV care platforms should be leveraged to reach HIV-negative women, and HPV testing should be integrated within primary care and maternal or child health services. In addition, reminder, recall, and navigation systems should be expanded beyond HIV clinics. SDOH barriers should be addressed directly through transport support, mobile screening for rural communities, flexible clinic hours, and help to ensure linkage from screening to diagnosis and treatment. Age-tailored outreach for HIV-negative women, including community and workplace campaigns focused on younger age groups, is recommended. Screening within HIV care should be maintained and standardized by scheduling offers and tracking re-screening intervals. A complementary qualitative study is underway to examine women’s lived experiences with cervical cancer screening, including stigma, health system navigation, and provider-patient interactions, with particular attention to differences by HIV status. These data will inform the design of targeted, context-specific interventions to improve screening uptake beyond what can be captured through quantitative SDOH measures alone.

## Supplementary Material

Supplementary Files

This is a list of supplementary files associated with this preprint. Click to download.

- SupplementaryFile1.pdf
- SupplementalTable1.docx

## Figures and Tables

**Table 1:** Social Determinants of Health Score (SDOH) Items and Definitionsa

SDOH Item	Definition and Score	
	0	1
**Economic stability**		
Do you have any source of income?	Yes	No
How hard is it for you to pay for the very basics—things like food, housing, and water?	Not hard at all	Somewhat hard/Very hard
**Education**		
Level of education	Secondary/Tertiary	None/Primary
Knowledge of cervical cancer	Yes	No
**Health/Substance Use**		
Do you consume alcohol?	No	Yes
Do you use tobacco?	No	Yes
**Neighborhood and built environment**		
Rurality	Urban area (Town)	Rural area (Village, Farm)
Housing instability (Score 0–7)	No	Yes
What is the type of housing unit?	Commercial building/Conventional flat	Traditional (Mud/Thatch)
What is the main type of material used for the roof?	Iron sheets	Thatch/Grass/Wood/Bamboo/Asbestos
What is the source of drinking water for your household members?	Piped water/Bottled water/Protected well	Borehole/Unprotected well/Surface water
What is the main source of energy used for cooking?	Electricity/Solar power/Gas	Charcoal/Wood
Where is the cooking usually done?	In the house	In a separate building/Outside
Problems with pests such as bugs, ants, mice, lead paints, water leak?	No	Yes
Problems with electricity?	No	Yes
**Social and Community Context**		
Are you able to do your day-to-day activities on your own?	Yes	No
Do you ever feel lonely or isolated from those around you?	No	Yes

a Based on questionnaire modified from the US Center for Medicare and Medicaid Services.

**Table 2:** Sociodemographic Characteristics of Participants by Prior Cervical Cancer Screening Status (N = 464)

Characteristic^a^	Overall N = 464		Prior Cervical Cancer Screening				*p*-value*
			No n = 249		Yes n = 215		
Age, y, median (IQR)	50 (43–57)		51 (42–60)		49 (43–55)		0.050
Age, y							0.001
29–39	74	15.9	41	55.4	33	44.6	
40–49	151	32.5	74	49.0	77	51.0	
50–59	150	32.3	70	46.7	80	53.3	
60–78	89	19.2	64	71.9	25	28.1	
Marital status							0.112
Single/separated/divorced/widowed	204	44.0	101	49.5	103	50.5	
Married	260	56.0	148	56.9	112	43.1	
HIV status							<0.001
Negative	213	45.9	145	68.1	68	31.9	
Positive	251	54.1	104	41.4	147	58.6	
Cancer stage							0.017
I/II	206	53.6	101	49.0	105	51.0	
III/IV	178	46.4	109	61.2	69	38.8	
Missing	80		39		41		
Province							0.245
Central	53	11.4	34	64.2	19	35.8	
Copperbelt	79	17.0	43	54.4	36	45.6	
Eastern	65	14.0	40	61.5	25	38.5	
Lusaka	133	28.7	65	48.9	68	51.1	
Southern	73	15.7	39	53.4	34	46.6	
Other	61	13.1	28	45.9	33	54.1	

a Data are no. (%) unless otherwise indicated.

HIV, human immunodeficiency virus

* p-values are based on the Wilcoxon rank-sum test for continuous variables and chi-squared test or Fisher exact test for categorical variables. *p* < 0.05 was considered statistically significant.

**Table 3:** Social Determinants of Health by Prior Cervical Cancer Screening Status (N = 464)

Characteristic^a^	Overall N = 464		Prior Cervical Cancer Screening				*p*-value*
			No n = 249		Yes n = 215		
**SDOH score**, median (IQR)	5 (4–6)		5 (4–6)		4 (3–6)		< 0.001
**SDOH score**							< 0.001
0–4, lower disadvantage	286	61.6	76	40.9	110	59.1	
5–10, higher disadvantage	178	38.4	173	62.2	105	37.8	
**Economic stability**							
Do you have any source of income?							0.758
No	141	30.5	74	52.5	67	47.5	
Yes	322	69.5	174	54.0	148	46.0	
How hard is it for you to pay for the very basics—things like food, housing, and water?							0.691
Not hard at all	45	9.8	23	51.1	22	48.9	
Somewhat hard/very hard	415	90.2	225	54.2	190	45.8	
**Education**							
Level of education							< 0.001
None/Primary	323	69.8	196	60.7	127	39.3	
Secondary/Tertiary	140	30.2	52	37.1	88	62.9	
Knowledge of cervical cancer							< 0.001
No	320	69.0	212	66.2	108	33.8	
Yes	144	31.0	37	25.7	107	74.3	
**Health/Substance Use**							
Do you consume alcohol?							0.117
No	353	76.2	197	55.8	156	44.2	
Yes	110	23.8	52	47.3	58	52.7	
Do you use tobacco?							0.518
No	446	96.1	238	53.4	208	46.6	
Yes	18	3.9	11	61.1	7	38.9	
**Neighborhood and built environment**							
Rurality							0.002
Rural area (Village, Farm)	224	48.4	137	61.2	87	38.8	
Urban area (Town)	239	51.6	111	46.4	128	53.6	
Housing instability							0.515
No	232	50.0	121	52.2	111	47.8	
Yes	232	50.0	128	55.2	104	44.8	
**Social and Community Context**							
Are you able to do your day-to-day activities on your own?							0.022
No	125	26.9	78	62.4	47	37.6	
Yes	339	73.1	171	50.4	168	49.6	
Do you ever feel lonely or isolated from those around you?							0.886
No	128	27.6	68	53.1	60	46.9	
Yes	336	72.4	181	53.9	155	46.1	

a Data are no. (%) unless otherwise indicated.

* p-values are based on the Wilcoxon rank-sum test for continuous variables and chi-squared test or Fisher exact test for categorical variables. *p* < 0.05 was considered statistically significant.

**Table 4:** Association of Prior Cervical Cancer Screening with Sociodemographic Characteristics and Social Determinants of Health

	Unadjusted models^a^ (*p* < 0.05)				Adjusted full model^b^ (*p* < 0.1)			
	OR	95% CI		*p*-value	OR	95% CI		*p*-value
Age, y								
50–59	Ref.							
21–39	0.70	0.40	1.23	0.219	0.67	0.37	1.20	0.178
40–49	0.91	0.58	1.43	0.685	0.83	0.51	1.33	0.436
60–78	0.34	0.19	0.60	< 0.001	0.41	0.23	0.73	0.003
HIV								
Negative	Ref.							
Positive	3.01	2.06	4.42	< 0.001	2.72	1.83	4.05	< 0.001
SDOH indicators								
5–10	Ref.							
0–4	2.38	1.63	3.48	< 0.001	2.21	1.48	3.28	< 0.001

OR = odds ratio, CI = confidence interval, Ref = reference group.

a Unadjusted models: univariable logistic regression models including variables with a p-value ≤ 0.05.

b Adjusted model: Logistic regression model using a stepwise backward elimination method with a p-value ≤ 0.10.

**Table 5:** Sociodemographic Characteristics of Participants by HIV Status (N = 464)

Characteristic^a^	Overall N = 464		HIV status				*p*-value*
			Negative n = 213		Positive n = 251		
Age, y, median (IQR)	50 (43–57)		52 (43–60)		48 (42–54)		0.003
Age, y							0.002
29–39	74	15.9	33	44.6	41	55.4	
40–49	151	32.5	57	37.7	94	62.3	
50–59	150	32.3	67	44.7	83	55.3	
60–78	89	19.2	56	62.9	33	37.1	
Marital status							<0.001
Single/separated/divorced/widowed	204	44.0	71	34.8	133	65.2	
Married	260	56.0	142	54.6	118	45.4	
Cancer stage							0.227
I/II	206	53.6	88	42.7	118	57.3	
III/IV	178	46.4	87	48.9	91	51.1	
Missing	80		38		42		
Province							0.027
Central	53	11.4	29	54.7	24	45.3	
Copperbelt	79	17.0	30	38.0	49	62.0	
Eastern	65	14.0	37	56.9	28	43.1	
Lusaka	133	28.7	49	36.8	84	63.2	
Southern	73	15.7	38	52.1	35	47.9	
Other	61	13.1	30	49.2	31	50.8	

a Data are no. (%) unless otherwise indicated

IQR, interquartile range

* p-values are based on the Wilcoxon rank-sum test for continuous variables and chi-squared test or Fisher exact test for categorical variables. *p* < 0.05 was considered statistically significant.

**Table 6:** Association of Prior Cervical Cancer Screening with Sociodemographic Characteristics and Social Determinants of Health Stratified by HIV Status

	HIV negative								HIV positive							
	Unadjusted models^a^ (*p* < 0.05)				Adjusted full model^b^ (*p* < 0.1)				Unadjusted models^a^ (*p* < 0.05)				Adjusted full model^b^ (*p* < 0.1)			
	OR	95% CI		*p*-value	OR	95% CI		*p*-value	OR	95% CI		*p*-value	OR	95% CI		*p*-value
Age, y																
50–59	Ref.															
29–39	0.52	0.20	1.37	0.189	0.37	0.14	0.99	0.049	0.70	0.31	1.62	0.410	0.88	0.4	1.95	0.751
40–49	0.67	0.31	1.49	0.330	0.82	0.38	1.77	0.612	0.75	0.39	1.42	0.370	0.85	0.45	1.59	0.608
60–78	0.28	0.12	0.68	0.005	0.31	0.13	0.78	0.012	0.48	0.21	1.13	0.095	0.43	0.18	1.00	0.050
Province																
Central	Ref.															
Copperbelt	2.22	0.69	7.12	0.180	1.77	0.51	6.18	0.372	0.88	0.33	2.35	0.800	0.75	0.27	2.07	0.575
Eastern	0.89	0.26	3.02	0.858	0.74	0.21	2.64	0.644	1.52	0.5	4.64	0.459	1.69	0.53	5.43	0.377
Lusaka	2.43	0.84	7.05	0.103	1.72	0.55	5.37	0.352	1.18	0.48	2.95	0.716	1.04	0.4	2.72	0.928
Southern	1.56	0.5	4.88	0.443	1.29	0.39	4.22	0.678	1.62	0.56	4.7	0.373	1.85	0.61	5.59	0.274
Other	3.35	1.06	10.59	0.039	3.77	1.11	12.88	0.034	1.34	0.45	3.95	0.596	1.35	0.44	4.13	0.598
Marital status																
Single/separated/divorced/widowed	Ref.															
Married	1.20	0.62	2.30	0.593	1.27	0.61	2.67	0.523	0.72	0.42	1.23	0.234	0.57	0.33	0.97	0.040
SDOH score																
5–10	Ref.															
0–4	2.26	1.20	4.28	0.012	2.18	1.13	4.17	0.019	1.86	1.08	3.21	0.024	2.15	1.25	3.69	0.006

* OR = odds ratio, CI = confidence interval, Ref = reference group.

a Unadjusted models: univariable logistic regression models including variables with a *p*-value ≤ 0.05.

b Adjusted model: Logistic regression model using a stepwise backward elimination method with a *p*-value ≤ 0.10.

## Data Availability

CDH’s being the only radiation oncology center in Zambia increases the risk of re-identification of patients. All relevant summary statistics are provided in the results section, and the survey appears in the supplemental methods to help clarify the data characteristics. Further data may be shared upon reasonable request.

## Abbreviations

CDH: Cancer Disease Hospital
CI: confidence interval
HPV: human papillomavirus
IQR: interquartile range
LMICs: low- and middle-income countries
OR: odds ratio
VIA: visual inspection with acetic acid
WHO: World Health Organization
WLHIV: women living with HIV
